# 

**Published:** 1898-02

**Authors:** 


BOOKS RECEIVED.

A Manual of Obstetrics. By A. F. A. King, M. D., Professor of Obstetrics and Diseases of Women in the Medical Department of the Columbian University, Washington, D. C., and in the University of Vermont, etc. New (seventh) edition. In one 12mo volume of 573 pages, with 223 illustrations. Cloth, $2.50. Lea Brothers & Co., Publishers, Philadelphia and New York. 1898.

Elements of Latin. For students of medicine and pharmacy. By George D. Crothers, A. M., M. D., Teacher of Latin and Greek in the St. Joseph (Mo.) High School; and Hiram H. Bice, A. M., Instructor in Latin and Greek in the Boys' High School of New York City. Pages xii.—242. Cloth, $1.25 net. Philadelphia, New York City, Chicago: The F. A. Davis Co., Publishers.

Outlines of Rural Hygiene. For Physicians, Students and Sanitarians. By Harvey B. Bashore, M. D., Inspector for the State Board of Health of Pennsylvania. With an appendix on The Normal Distribution of Chlorine, by Prof. Herbert E. Smith, of Yale University. Illustrated with twenty (20) engravings. Pages vi.—84. Cloth, 75 cents net. Philadelphia, New York City, Chicago: The F. A. Davis Co., Publishers.

Proceedings of the United States Veterinary Medical Association. Session of 1897. Thirty-fourth annual meeting held at Nashville, September 7, 8 and 9, 1897. Edited by the publication committee, W. L. Williams, Chairman, Cornell University, Ithaca, N. Y.

Transactions of the American Microscopical Society. Twentieth annual meeting, held at Toledo, O., August 5, 6 and 7, 1897. William C. Krauss, M. D., Secretary.

Transactions of the National Confederation of State Medical Examining and Licensing Boards. Seventh annual meeting, held at Philadelphia, Pa., May 31, 1897. A. Walter Suiter, M. D., Secretary.

Transactions of the Oregon State Medical Society. Twenty-fourth annual meeting, held at Portland, Oregon, June 8 and 9, 1897. William F. Amos, M. D., Secretary.

